# Fe-EDTA-*Bis*amide and Fe-ADR-925, The Iron-Bound Hydrolysis Product of the Cardioprotective Agent Dexrazoxane, Cleave DNA Via the Hydroxyl Radical

**DOI:** 10.1155/MBD.1997.199

**Published:** 1997

**Authors:** Thomas J. Magliery, Lizabeth K. Vitellaro, Ndeye Khady Diop, Rosemary A. Marusak

**Affiliations:** 1 Department of Chemistry Kenyon College Gambier OH 43022 USA; 2 Department of Chemistry University of California Berkeley CA 94720 USA

## Abstract

Use of the antitumor drug doxorubicin is limited by cardiomyopathic side-effects which are believed to be due to iron-mediated hydroxyl radical generation. Dexrazoxane reduces this cardiotoxicity, possibly by removal of iron from doxorubicin by the EDTA-like hydrolysis product of dexrazoxane, ADR-925. However, EDTA-diimides like dexrazoxane, previously used as antitumor agents, are themselves carcinogenic, and recent studies have found that Fe-ADR-925 can also promote hydroxyl radical production. This study demonstrates that, like Fe-EDTA, Fe-ADR-925 and a related desmethyl complex can cleave plasmid DNA under Fenton conditions, and suggests by radical scavenger study that this cleavage is probably via the hydroxyl radical. Differences in DNA cleavage dependence upon concentrations of Fe-EDTA, Fe-ADR-925 and Fe-EDTA-*bis*amide can be explained by differences in the solution chemistry of the complexes.


### Fe-EDTA-*BIS*AMIDE AND Fe-ADR-925, THE IRON-BOUND HYDROLYSIS PRODUCT OF THE CARDIOPROTECTIVE AGENT DEXRAZOXANE, CLEAVE DNA VIA THE HYDROXYL RADICAL

Thomas J. Magliery,[#] Lizabeth K. Vitellaro, Ndeye Khady Diop and Rosemary A. Marusak*

Department of Chemistry, Kenyon College, Gambier, OH 43022, USA

**Abstract**

Use of the antitumor drug doxorubicin is limited by cardiomyopathic side-effects which are believed to be due to iron-mediated hydroxyl radical generation. Dexrazoxane reduces this cardiotoxicity, possibly by removal of iron from doxorubicin by the EDTA-like hydrolysis product of dexrazoxane, ADR-925. However, EDTA-diimides like dexrazoxane, previously used as antitumor agents, are themselves carcinogenic, and recent studies have found that Fe-ADR-925 can also promote hydroxyl radical production. This study demonstrates that, like Fe-EDTA, Fe-ADR-925 and a related desmethyl complex can cleave plasmid DNA under Fenton conditions, and suggests by radical scavenger study that this cleavage is probably via the hydroxyl radical. Differences in DNA cleavage dependence upon concentrations of Fe-EDTA, Fe-ADR-925 and Fe-EDTA-*bis*amide can be explained by differences in the solution chemistry of the complexes.

**Introduction**

Dexrazoxane (Zinecard, Cardioxane, ADR-529, ICRF-187), **1**, reduces the cardiomyopathic effects of the antitumor antibiotic doxorubicin (Adriamycin), effectively raising the lifetime maximum dose of doxorubicin.[1] The cardiotoxicity of doxorubicin is believed to be due to an iron-mediated production of reactive hydroxyl radicals, HO•, which have been associated with lipid peroxidation and protein and nucleic acid cleavage.[2,3] It is generally thought that the nonpolar dexrazoxane hydrolyzes intracellularly to **2** (ADR-925), a polar *bis*amide/*bis*acid analog of the metal ion chelator EDTA (**3**), reducing the availability of iron to doxorubicin and, hence, the production of hydroxyl radicals.[2] In addition to the enantiomerically-pure dexrazoxane, both its racemate razoxane (ICRF-159) and its desmethyl form (ICRF-154, EDTA-diimide, **4**), are alone effective against murine tumor models.[4,5] Razoxane was previously used to treat breast masses as well as psoriasis, but treatment with EDTA-diimide derivatives led to generalized carcinogenesis as therapy-related leukemia.[5,6] Like doxorubicin, razoxane is thought to target topoisomerase II (although by a different mechanism); however it was shown to hydrolyze intracellularly and was initially thought to be involved in metal ion chelation.[7-11]

Dexrazoxane is prescribed at lower doses in cardioprotection (500-600 mg/m$^2$) than razoxane was as an antineoplastic (500-1500 mg/m$^2$), but an important therapeutic issue is the limit of the ability of dexrazoxane to reduce doxorubicin-related toxicity, especially since razoxane was shown to act synergistically with doxorubicin as an antitumor agent.[5,12] Indeed, Imondi et al. recently showed that proportionally greater doses of dexrazoxane are poorer inhibitors of cardiomyopathy at increasing doses of doxorubicin in animal models.[13] Hasinoff and Winterbourn have illustrated the ability of the hydrolysis product of razoxane (ICRF-198, the racemate of ADR-925) to remove iron(III) from doxorubicin, and there is recent evidence that the iron-bound hydrolysis product of dexrazoxane is involved in hydroxyl-radical production.[14,15] Winterbourn, et al., illustrated this production by showing oxidation of small, organic molecules in the presence of Fe-ADR-925 comparable to Fe-EDTA.[15] Hasinoff and associates have shown that this can involve direct reaction of doxorubicin to reduce the Fe-ADR-925 and have additionally suggested that Fe-ADR-925 produces hydroxyl radicals more slowly than Fe-EDTA.[16,17]

[#]Current Address: Department of Chemistry, University of California, Berkeley, CA 94720, USA

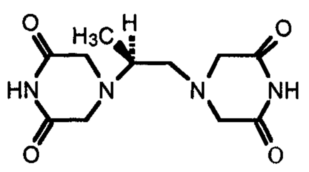

**1** dexrazoxane

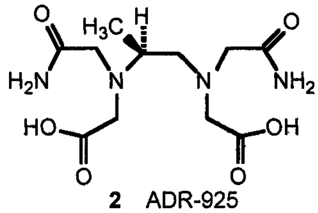

**2** ADR-925

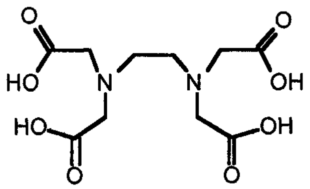

**3** EDTA

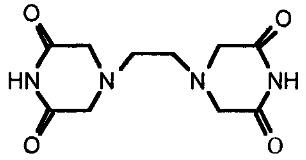

**4** ICRF-154

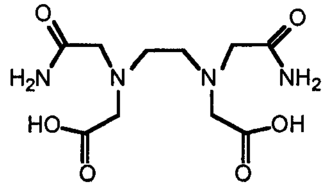

**5** EDTA-*bis*amide

Fe-ADR-925 is presumed to react like Fe-EDTA under Fenton conditions (in the presence of oxidant and reductant).[15] The Fenton reaction involves reduction of the ferric species to the corresponding ferrous species, followed by reduction of dioxygen to hydrogen peroxide or reduction of hydrogen peroxide to a hydroxide and hydroxyl radical; other electron-deficient species may also be produced, depending upon the iron ligand.[3] In particular, these reactive, oxidizing species are exploited in nucleic acid and protein cleavage studies, and it has specifically been illustrated that Fe-EDTA cleaves DNA under Fenton conditions via the hydroxyl radical.[3,18]

Since doxorubicin is a DNA intercalator, it is reasonable to think that some of the hydroxyl radicals produced by the doxorubicin oxidize and cleave DNA *in vivo*, and this may account in part for the cardiotoxicity of doxorubicin. However, since Fe-ADR-925 produces hydroxyl radicals similarly to Fe-EDTA, it is of great importance to consider whether this iron-bound hydrolysis product of dexrazoxane can also cleave DNA. A particularly useful assay for hydroxyl radical damage to DNA is the nicking of a supercoiled plasmid.[19-23] The supercoiled form (form I) of the plasmid can readily be separated from plasmid that has been cleaved in a single-stranded manner (nicked, form II) and in a double-stranded manner (linear, form III) by agarose gel electrophoresis. This study investigates the ability of iron-bound ADR-925 and its desmethyl derivative EDTA-*bis*amide (**5**) to cleave supercoiled plasmid DNA in the presence of the reductant ascorbic acid under aerobic conditions. Additionally, thiourea, known to be a radical scavenger, is used to probe the mechanism of DNA cleavage.

### Materials and Methods

*Materials.* EDTA-dianhydride, thiourea, $Na_2Fe^{III}EDTA\Sigma 2H_2O$, aqueous glycerol and boric acid (Aldrich); ascorbic acid and $FeCl_2$ (Baker); Tris and bromphenol blue (BioRad); conc. $NH_4OH$, NaCl and $Na_2EDTA$ (Fisher); agarose (EEO 0.9-0.13) and ethidium bromide (Sigma) were obtained in their highest available purity and used without further purification. ADR-925 (**2**) was generously provided by Pharmacia/Upjohn. Universal buffer was from Stratagene, and EcoR I and pBR322 plasmid solution (0.25μg/μL, 10 mM Tris HCl, 5 mM NaCl, 0.1 mM EDTA, pH 7.4) were from GibcoBRL. Barnsted Nanopure (18 MΩ cm) water was used throughout, and all reaction equipment was acid-washed in 4.5 M HCl or concentrated sulfuric acid, or purchased metal-free.

*Instrumentation.* NMR spectra were obtained using a Varian Gemini 2000 200 MHz spectrometer equipped with a Sun Workstation. UV-VIS spectra were acquired on an Olis Cary-14 spectrophotometer interfaced to a Dell 310 microcomputer. IR spectra were obtained using a Perkin Elmer 1600 Series FTIR spectrophotometer. Solution sample and reaction pH were monitored with a Corning Model 340 pH meter equipped with a Corning combination electrode. Electrophoresis was carried out in a Submarine Mini-gel Electrophoresis Unit (Sigma) with a BioRad PowerPac 300. Gels were visualized with an IS-1000 Digital Imaging System (Alpha Innotech Corp.).

*Preparation of EDTA-bisamide (5).* Ethylenediaminetetraacetic acid dianhydride (6.60 g, 25.8 mmol) was slowly stirred into an ice-cold 20 mL portion of concentrated (28-30%) ammonium hydroxide. At room temperature, an additional 1 mL of conc. ammonium hydroxide was added, followed by 20 mL of water and 12-24 h of stirring. Solvent was then removed under reduced pressure, and the white solid was redissolved in 20 mL water and precipitated with concentrated HCl. This was filtered under vacuum, washed twice with dry diethyl ether and vacuum desiccated overnight. Analysis: yields >75%; IR carbonyl bands (KBr): 1645.8 $cm^{-1}$ ($CONH_2$) and 1680.4 $cm^{-1}$ (COOH); $^1H$ NMR ($D_2O$; DSS internal standard) resonances: 2.9 ppm (N-$CH_2$), 3.35 ppm ($CH_2CO_2H$), 3.5 ppm ($CH_2CONH_2$).

*Preparation and Characterization of Iron Complexes.* Fe-EDTA was used as purchased. Fe-EDTA-*bis*amide and Fe-ADR-925 were freshly-prepared 10× *in situ* by mixture of appropriate amounts of EDTA-*bis*amide or ADR-925 (0.2 M stock) and ferrous chloride (0.04 M stock) and maintained at pH 5. Resulting solutions were diluted into water for immediate use. EDTA-*bis*amide and ADR-925 were used at 500 μM regardless of iron concentration, which was at least in 4-fold excess over the iron complex. In aqueous solution, these complexes exist in both monomeric and dimeric forms. At pH 5, the iron(III) EDTA-*bis*amide and ADR-925 monomers show one absorbance maximum at 258 nm. The dimeric complexes (pH 7) exhibit shoulders at 247 nm, 271 nm, 308 nm, and 341 nm and a maximum in the visible region at 475 nm and 540 nm (sh).

Base titrations were carried out at 23 °C and over pH ranges 4.0-6.9 and 4.2-8.2 for Fe-EDTA-*bis*amide and Fe-ADR-925, respectively. At higher pH, the iron complexes become unstable towards precipitation. Iron(III) complexes (100 μM) were generated *in situ* by the addition of 1:1 ferrous chloride to a stock solution of the ligand (pH~5) and aerially oxidized to the iron(III) form over approximately 24 hrs. Throughout the titration, standardized, carbonate-free NaOH(aq) (0.1 M) was added with continuous stirring and under a blanket of $N_{2(g)}$ to raise the pH approximately 0.2 units. Spectra and pH readings were obtained 10 minutes after each addition of base. During this 10 min. interval, the pH drops and minimizes; kinetics of similar pH-jump experiments indicate dimer formation maximizes in 10 min., after which time a slower secondary process (most likely re-equilibration with the monomer species) takes place. Titrations at higher metal-complex concentrations, needed for monitoring dimer formation at 475 nm, were unsuccessful due to precipitation.

*Nicking Reactions.* Each 20 μL reaction was 10.2 mM Tris HCl, 50.4 mM NaCl, 2.5 μM EDTA, 0.1 mM ascorbate, 10 μM in base pairs of DNA, specified concentration of iron reagent and excess ligand, pH 7. The DNA used was the pBR322 plasmid from *E. coli*, which is 4,361 base pairs and has a single, unique recognition site for the EcoR I endonuclease (which cleaves DNA in a double-stranded manner). Tris/NaCl buffer (pH 7) with thiourea when appropriate (17.5 μL) and DNA solution (0.5 μL) were combined in a picofuge tube; 1.0 μL of freshly-prepared ascorbate (2 mM, pH 7) and 1.0 μL of freshly-prepared iron-reagent solution were placed on the side of the tube, and all reactions for each experiment were started nearly simultaneously by shaking the components of each tube together. Reactions proceeded at room temperature for 60 min, and each was stopped by the addition of 5 μL of a solution of 0.25% bromphenol blue in 30% aqueous glycerol and electrophoresed immediately.

*(A)* To confirm the relative electrophoretic mobilities of plasmid forms I, II and III, reactions with no iron reagent, with 100 μM ferrous chloride and with a standard 20 μL EcoR I digest were performed. *(B)* Fe-EDTA was studied at 1 μM, 10 μM, and 100 μM in the absence of thiourea. *(C)* Fe-EDTA-*bis*amide was studied at 1 μM, 10μM, 50 μM and 100 μM in the absence of thiourea. *(D)* Fe-ADR-925 was studied at 10 μM, 50 μM and 100 μM in the absence of thiourea. *(E)* In the presence of 5 mM and 500 mM thiourea, Fe-EDTA and Fe-EDTA-*bis*amide were studied at 10 μM.

For cleavage product analysis, gels were 1% agarose in 0.5× TBE (45 mM Tris-borate, 1 mM EDTA, pH 7.75), and electrophoresis was conducted at room temperature in 0.5× TBE at 50 V for 3.5 h. After electrophoresis, gels were stained in 0.5 μg/mL ethidium bromide for 1 h and then imaged and analyzed densitometrically under ultraviolet light. Note that small differences in staining make exact quantitative gel-to-gel comparisons difficult. Densitometry is uncorrected for differential uptake of ethidium bromide by supercoiled and non-supercoiled DNA. A previous study with pBR322 plasmid under similar conditions showed this factor was small.[24]

**Results of DNA Cleavage Experiments**

Controls indicated that plasmid not treated with iron reagent or endonuclease was at least 80% supercoiled. Treatment with 100 μM ferrous chloride in the absence of ligand resulted in two bands of approximately equal densitometric intensity (the faster-migrating corresponding to the 80+% band on the control), and endonuclease digestion resulted in a single intermediate band. This indicates that under the electrophoretic conditions chosen, supercoiled DNA migrates the fastest, linear DNA migrates intermediately, and nicked DNA migrates most slowly.

**Table I.** Cleavage of pBR322 Plasmid With Varying Concentrations of Fe-EDTA and Fe-EDTA-*bis*amide.

| iron reagent | conc. (μM) | form (%) I | II | III |
|---|---|---|---|---|
| $Fe^{2+}$ | 100 | 35 | 64 | 1 |
| Fe-EDTA | 100 | 0 | 94 | 6 |
| Fe-EDTA | 10 | 3 | 88 | 9 |
| Fe-EDTA | 1 | 25 | 69 | 6 |
| Fe-EDTA*ba* | 100 | 10 | 84 | 6 |
| Fe-EDTA*ba* | 10 | 3 | 87 | 10 |
| Fe-EDTA*ba* | 1 | 41 | 59 | 0 |

Relative densitometric intensities from UV imaging after ethidium bromide staining. DNA (10 μ M bp), buffer (10 mM Tris HCl, 50 mM NaCl, 2.5 μ M EDTA, pH 7), ascorbate (100 mM) and iron reagent reacted at room temperature for 60 min.

**Table II.** Cleavage of pBR322 Plasmid With Fe-EDTA*bis*amide anc Fe-ADR-925.

| iron reagent | conc. (μM) | form (%) I | II | III |
|---|---|---|---|---|
| $Fe^{2+}$ | 100 | 30 | 70 | 0 |
| Fe-EDTA*ba* | 100 | 14 | 81 | 5 |
| Fe-EDTA*ba* | 50 | 7 | 88 | 5 |
| Fe-EDTA*ba* | 10 | 7 | 86 | 7 |
| Fe-ADR-925 | 100 | 4 | 91 | 5 |
| Fe-ADR-925 | 50 | 6 | 89 | 5 |
| Fe-ADR-925 | 10 | 13 | 82 | 5 |

Relative densitometric intensities fr UV imaging after ethidium bromide staining. Same conditions as in Ta

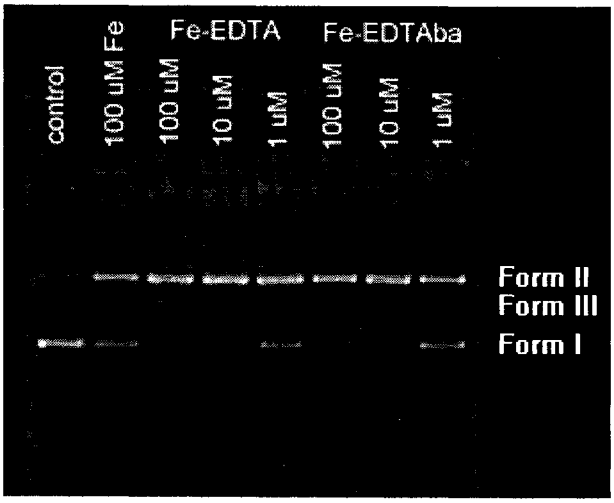

**Figure 1.** UV-visualized agarose gel of the pBR322 plasmid cleavage products after reaction with Fe-EDTA and Fe-EDTA-*bis*amide (1-100 μM). Supercoiled DNA is Form I, nicked is Form II, and linear is Form III.

DNA was cleaved by the Fe-EDTA-*bis*amide under Fenton conditions. At 100 μM, Fe-EDTA and Fe-EDTA-*bis*amide both cleaved the plasmid with greater efficiency than ferrous chloride (Table I). At 10 μM, Fe-EDTA and Fe-EDTA-*bis*amide cleaved the DNA with approximately equal efficiency. However, Fe-EDTA cleaved the DNA more efficiently than Fe-EDTA-*bis*amide at both 1 μM and 100 μM; this is especially unusual, since Fe-EDTA-*bis*amide cleaved the DNA less efficiently at 100 μM (the higher concentration) than at 10 μM (Figure 1). Fe-ADR-925 (the iron-bound hydrolysis product of dexrazoxane) was also able to cleave DNA (Table II), and did so with approximately equal efficiency to Fe-EDTA-*bis*amide at 50 μM. However, like Fe-EDTA, Fe-ADR-925 cleaves DNA with increasing efficiency as concentration increases between 10 μM and 100 μM (as opposed to Fe-EDTA-*bis*amide).

**Table III.** Thiourea Inhibition of Cleavage of pBR322 Plasmid by $Fe^{2+}$, Fe-EDTA and Fe-EDTA-*bis*amide.

| iron reagent | thiourea conc. (mM) | form (%) | | |
|---|---|---|---|---|
| | | I | II | III |
| $Fe^{2+}$ | 0 | 49 | 51 | 0 |
| $Fe^{2+}$ | 5 | 81 | 19 | 0 |
| Fe-EDTA | 0 | 0 | 97 | 3 |
| Fe-EDTA | 5 | 53 | 47 | 0 |
| Fe-EDTA | 500 | 92 | 8 | 0 |
| Fe-EDTA*ba* | 0 | 0 | 95 | 5 |
| Fe-EDTA*ba* | 5 | 48 | 52 | 0 |
| Fe-EDTA*ba* | 500 | 88 | 12 | 0 |

Relative densitometric intensities from UV imaging after ethidium bromide staining. Iron reagent is 10 μM. Other conditions same as Table 1.

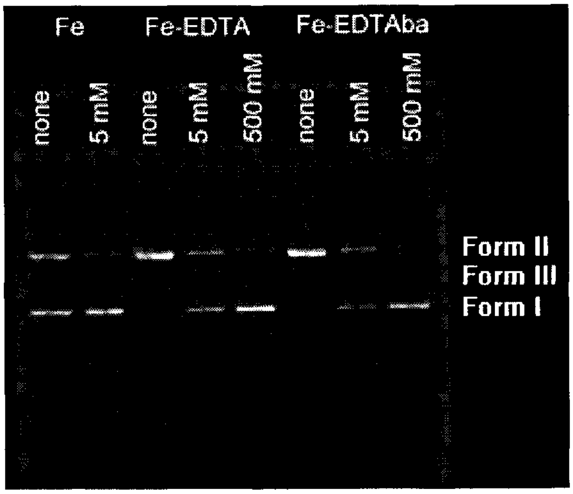

**Figure 2** Thiourea inhibition of DNA cleavage by $Fe^{2+}$, Fe-EDTA and Fe-EDTA-*bis*amide. Iron reagents were 10 μM, and thiourea was 0-500 mM (thiourea concentration is indicated over each lane). Same visualization as in Figure 1.

The cleavage of DNA in the presence of ferrous chloride, Fe-EDTA and Fe-EDTA-*bis*amide (all at 10 μM) was inhibited by the radical scavenger thiourea (Table III). At 5 mM thiourea, cleavage by ferrous chloride was almost entirely retarded, and cleavage by Fe-EDTA and Fe-EDTA-*bis*amide was reduced by half. Thiourea at 500 mM approximately completely prevented DNA cleavage (Figure 2).

### Discussion

Razoxane, like other EDTA-diimide drugs (ICRF-154, dexrazoxane), is an antineoplastic, and this is probably due at least in part to inhibition of topoisomerase II.[5,7] At lower doses, dexrazoxane, which is simply enantiomerically-pure razoxane, ameliorates the cardiotoxicity of doxorubicin therapy.[1] However, such diimides are also carcinogenic, and both razoxane and dexrazoxane hydrolyze *in vivo* to metal-ion chelators that can produce hydroxyl radicals when bound to iron under Fenton conditions.[6,10,15] This study illustrates that this chemistry can cleave DNA, and further suggests that this DNA cleavage is by hydroxyl radical, since it is inhibited by the radical scavenger thiourea.

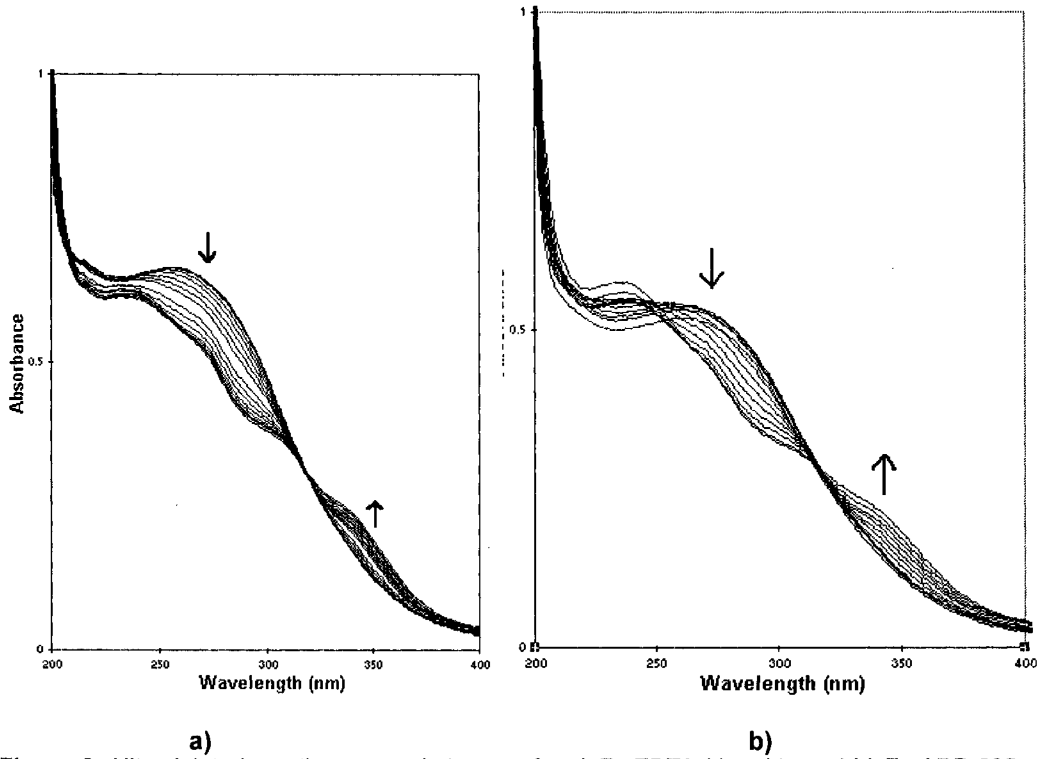

**Figure 3** Ultraviolet absorption spectral changes for **a)** Fe-EDTA-*bis*amide and **b)** Fe-ADR-925 upon base titration. Iron complexes were 100 μM. The pH ranges were 4.0-6.9 for Fe-EDTA-*bis*amide and 4.2-8.2 for Fe-ADR-925. At higher pHs, the iron complexes become unstable toward iron hydroxide and oxide formation and precipitation.

Our lab has observed that high (preparative) concentrations of ferrous chloride and EDTA-*bis*amide result in an initially yellow solution that turns brown over time at relatively low pH. UV studies have indicated that this is a secondary base-catalyzed reaction after complex formation, and spectrophotometric similarities between this reaction and formation of the Fe-EDTA μ-oxo dimer suggest that such μ-oxo dimerization may be occurring with Fe-EDTA-*bis*amide and Fe-ADR-925, as well (Figure 3). Dimer formation for Fe-ADR-925 begins at higher basicity (pH~6) than for Fe-EDTA-*bis*amide (pH~5), perhaps because the methyl substituent on ADR-925 decreases the Lewis acidity of the iron in that complex.[25] Attempts to determine dimerization constants by spectrophotometric titrations have been hindered by precipitation problems under the conditions needed for this analysis.[26] Further attempts to obtain these constants kinetically have likewise proven difficult due to mechanistic complexities. More work is needed, and a full analysis of this system is ongoing in our lab. The higher pHs at which Fe-EDTA and Fe-ADR-925 undergo monomer-dimer equilibrium, however, correspond well with the differential concentration/cleavage profiles of Fe- EDTA-*bis*amide, Fe-ADR-925 and Fe-EDTA. The inhibition of these DNA scission reactions by thiourea probably does not rule out either additional oxidizing species or modes of inhibition in addition to radical scavenging. In particular, thiourea might react with other electron-deficient species that could cleave DNA, and might also be binding to the metal center. The former is particularly worth further investigation since the ferryl species [$Fe^{IV}$=O(EDTA-*bis*amide)] would be neutral as opposed to [$Fe^{IV}$=O(EDTA)]$^{2-}$, which would be negative (same as the DNA backbone).

We would like to suggest that the ability of the iron-bound hydrolysis products of dexrazoxane and ICRF-154 to cleave DNA might account for the carcinogenic nature of the diimide drugs, and this implies a therapeutic limit to dexrazoxane. Furthermore, it does not seem clear at this time that such a route might not also be a substantial contributor to the *in*

*vivo* mechanism of the antitumor activity by razoxane. Most importantly, the production of the very same oxidizing species, HOΣ, that is taken to be responsible for doxorubicin toxicity at least suggests that there may be a maximum dose for doxorubicin/dexrazoxane combination. Moreover, the relative ease with which Fe-EDTA-*bis*amide and Fe-ADR-925 form dimers at near physiological pH suggests that the local concentration *in vivo* may be important to the iron-bound drug's overall reactivity. This calls for further chemical and, especially, clinical study.

**Acknowledgments**

The authors extend their gratitude to Dr. Anthony Imondi of Pharmacia/Upjohn for advice and samples of ADR-925, and to Professor David Marcey of Kenyon College. Additional thanks to Professors Thomas Tullius of Johns Hopkins University and A. Graham Lappin of the University of Notre Dame for helpful conversation relating to technical issues of this study and to Douglas Scheftner for preliminary work. Funding for this project was generously provided by a Robert J. Tomsich Award (Kenyon College, TJM and RAM) and a Research Corporation Award CC3833 (RAM).

**References**

1. Hellmann, K.; Franks, C.R. *Contrib. Oncol.* 1995, **48**, 40-47.
2. Doroshow, J.H. *ACS Symp. Ser. 574*, 1995, 259-267
3. Marusak, R.A.; Meares, C.F. *Active Oxygen in Biochemistry* (Valentine, J.S.; Foote, C.S.; Greenberg, A.; Liebman, J.F. Eds.) New York: Blackie Academic and Professional, 1995 pp 336-400.
4. Creighton, A.M.; Hellmann, K; Whitecross, S. *Nature* 1969, **222**, 384-385.
5. Herman, E.H.; Witiak, D.T.; Hellmann, K; Waravdekar, V.S. *Adv. Pharm. Chemo.* 1982, **19**, 249-290.
6. Xue, Y.; Lu, D.; Guo, Y.; Lin, B. *Leukemia Research* 1992, **16**, 1113-1123.
7. Tanabe, K.; Ikegami, Y.; Ishida, R.; Andoh, T. *Cancer Res.* 1991, **51**, 4903-4908.
8. Roca, J.; Ishida, R.; Berger, J.M.; Andoh, T.; Wang, J.C. *Proc. Natl. Acad. Sci. USA* 1994, **91**, 1781-1785.
9. Ishida, R.; Hamatake, M.; Wasserman, R.A.; Nitiss, J.L.; Wang, J.C.; Andoh, T. *Cancer Res.* 1995, **55**, 2299-2303.
10. Dawson, K.M. *Biochem. Pharm.* 1975, **24**, 2249-2253.
11. Albanese R.; Watkins, P.A. *Br. J. Cancer* 1985, **52**, 725-731.
12. Wadler, S.; Green, M.D.; Basch, R.; Muggia, R.M. *Biochem. Pharm.* 1987, **36**, 1495-1501.
13. Imondi, A.R.; Dellatorre, P.; Mazue, G.; Sullivan, T.M.; Robbins, T.L.; Hagerman, L.M.; Podesta, A.; Pinciroli, G. *Cancer Res.* 1996, **56**, 4200-4204.
14. Hasinoff, B.B. *Agents and Actions* 1989, **26**, 378-385.
15. Thomas, C.; Vile, G.F.; Winterbourn, C.C. *Biochem. Pharm.* 1993, **45**, 1967-1972.
16. Malisza, K.L.; Hasinoff, B.B. *Arch. Biochem. Biophys.* 1995, **316**, 680-688.
17. Hasinoff, B.B. *Free Rad. Res.* 1995, **22**, 319-325.
18. Pogozelski, W.K.; McNeese, T.J.; Tullius, T.D. *J. Am. Chem. Soc.* 1995, **117**, 6428-6433.
19. Hertzberg, R.P.; Dervan, P.B. *J. Am. Chem. Soc.* 1982, **104**, 313-315.
20. Iverson, B.L.; Shreder, K.; Morishima, T.; Rosingana, M.; Sessler, J.L. *J. Org. Chem.* 1995, **60**, 6616-6620.
21. Chang, C.-H.; Meares, C.F. *Biochemistry* 1982, **24**, 6332-6334.
22. Shepherd, R.E.; Lomis, T.J.; Koepsel, R.R.; Hegde, R.; Mistry, J.S. *Inorg. Chim. Acta* 1990, **171**, 139-149.
23. Morrow, J.R.; Kolasa, K.A. *Inorg. Chim. Acta* 1992, **195**, 245-248.
24. Hertzberg, R.P.; Dervan, P.B. *Biochemistry* 1984, **23**, 3934-3945.
25. Schugar, H.J.; Hubbard, A.T.; Andson, F.C.; Gray, H.B. *J. Am. Chem. Soc.* 1969, **90**, 71-77.
26. As defined by Sillén, L. G. *Quart. Rev.* (London), 1959, **13**, 146-168.

**Received: February 12, 1997 - Accepted: February 20, 1997 - Received in revised camera-ready format: May 26, 1997**

